# Liver Pseudotumor Due to Aberrant Left Gastric Vein: A Case Report

**DOI:** 10.5334/jbsr.3342

**Published:** 2023-10-27

**Authors:** Serhat Deger, Ahmet Bozer

**Affiliations:** 1 Department of Radiology, Bozyaka Educatıon and Research Hospıtal, İzmir, Turkey

**Keywords:** aberrant left gastric vein (ALGV), focal fatty sparing, liver, pseudotumor, third inflow

## Abstract

Liver pseudotumors (pseudolesions) pose diagnostic challenges in imaging, often linked to anomalous venous drainage. Notably, aberrant left gastric vein (ALGV) plays a role in segment 2 and 3 pseudolesions. A liver parenchymal pseudolesion due to abnormal venous drainage involving ALGV is highlighted. Understanding hepatic vascular dynamics, exemplified by ALGV-related pseudolesions, aids diagnosis and guides decisions. Further investigating intricate mechanisms underlying these anomalies is crucial.

**Teaching Point:**

The recognition of third inflow pathways such as ALGV holds significance in distinguishing hepatic pseudolesions from true lesions.

## Introduction

Liver pseudotumors, also known as pseudolesions, pose a significant diagnostic challenge in liver imaging. They are characterized by anomalous venous drainage into the liver, including aberrant right gastric venous drainage, parabiliary venous system, epigastric, and cholecystic veins. Among these, aberrant left gastric vein (ALGV) has been identified as a rare entity responsible for pseudolesions observed in liver segments 2 and 3 on post-contrast images [1].

By emphasizing the identification of ALGV-related pseudolesions, our goal is to enhance comprehension of this rare condition, aiding clinicians in precise diagnosis and effective management, supported by diagnostic imaging.

## Case Report

A 49-year-old male patient presented with non-specific symptoms, including abdominal pain and bloating. Physical examination revealed no specific findings except mild tenderness upon palpation in the right upper quadrant. Modest derangements in liver function parameters and increased lipid profiles were noted in the patient’s medical records. To elucidate the etiology of the symptoms, an abdominal ultrasound (USG) was conducted. Abdominal USG revealed a hypoechoic solid lesion measuring approximately 5 × 4 cm, situated within segments 2–3 of the hepatic parenchyma. This lesion was observed on a background of fatty liver tissue.

**Figure 1:**
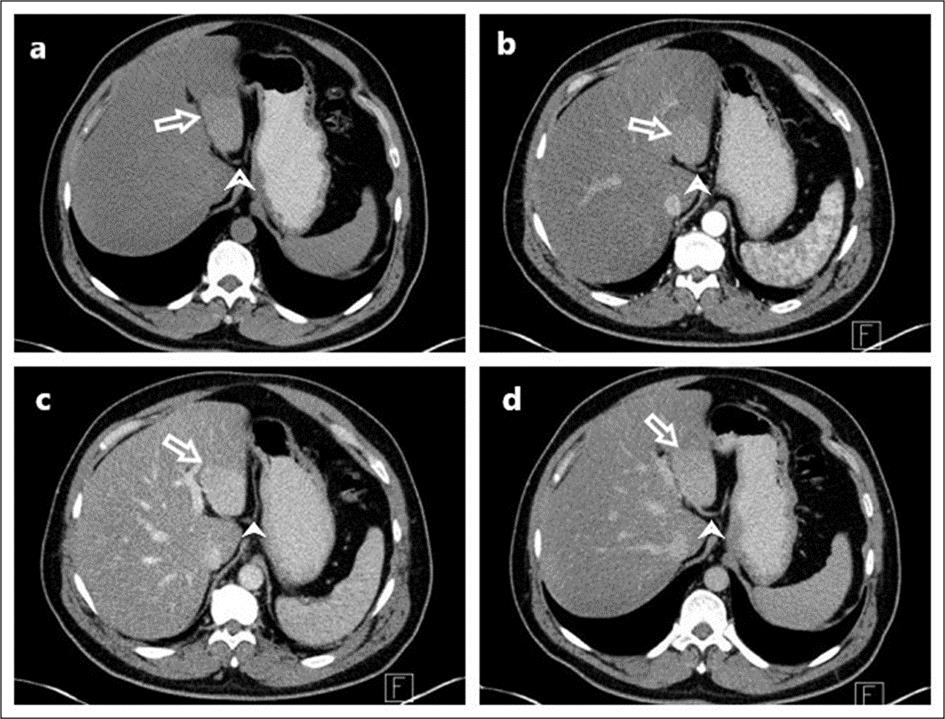
Pseudolesion in liver segments II and III associated with aberrant left gastric vein. axial (CT) images were acquired using a four-phase liver CT protocol: **(a)** pre-contrast, **(b)** arterial phase, **(c)** portal venous phase, and **(d)** delayed phase. These images vividly illustrate the presence of the pseudolesion (arrow) within liver segments II and III. The pseudolesion, showing heightened contrast enhancement compared to the surrounding parenchyma, is a result of the third inflow drainage of the aberrant left gastric vein (ALGV) (arrowhead). The characteristic entry site and course of ALGV are accurately delineated.

Subsequently, a dynamic contrast-enhanced abdominal computed tomography (CT) scan was conducted to provide a more comprehensive evaluation of the lesion. In this dynamic assessment, a hyperdense area was observed in liver segments 2–3, distinct from the surrounding hepatic parenchyma, both in the pre-contrast phase and subsequent phases. While the density of this area was higher than the surrounding parenchyma in the contrast phase, it was similarly contrast-enhanced, resembling the parenchyma. During the venous phase, it was noted that ALGV terminated within this area of the parenchyma. This pseudolesion appearance was attributed to abnormal venous drainage (Figure 1).

**Figure 2:**
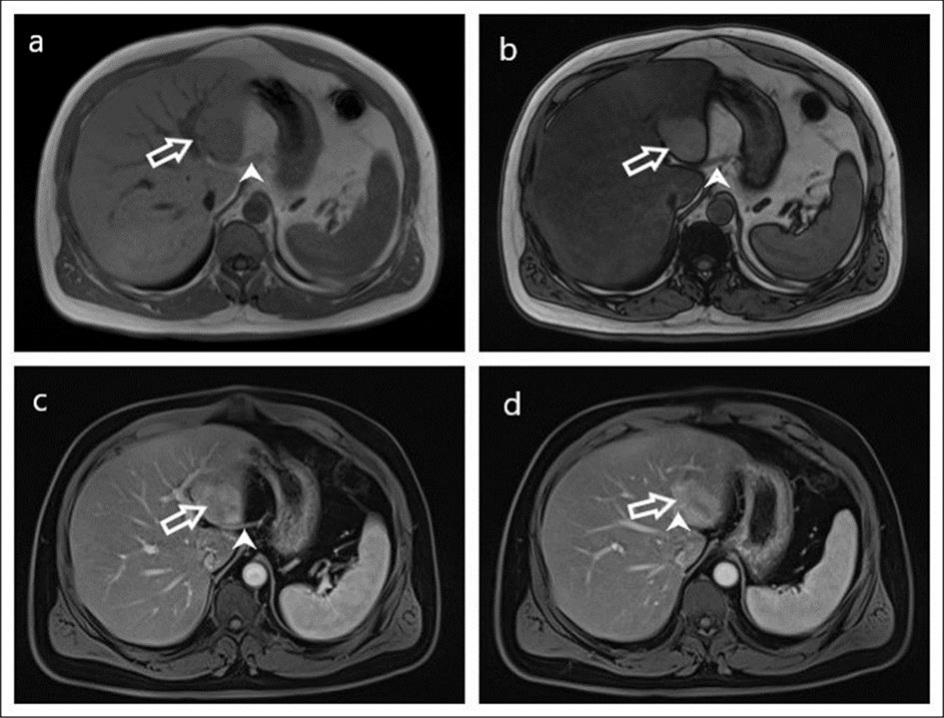
Pseudolesion in liver segments II and III associated with aberrant left gastric vein. MRI images, including in-phase **(a)** and out-of-phase **(b)**, reveal the pseudolesion (arrow) exhibiting compatibility with an area of focal fatty sparing. In the contrast-enhanced MRI, during the portal venous phase **(c, d)**, the entry and course of ALGV (arrowhead) are demonstrated within the parenchyma, consistent with Type 1 branching.

After this, the patient underwent dynamic magnetic resonance imaging (MRI) of the upper abdomen. The absence of signal loss in the out-of-phase magnetic resonance imaging (MRI) sequence supported the diagnosis of focal fatty sparing. In other sequences of the MRI, a distinct signal was observed within this area due to abnormal venous drainage, setting it apart from the surrounding parenchyma (Figure 2).

In this case, we advised lifestyle changes, including a balanced diet, exercise, medical supervision, medication as needed, and avoiding alcohol. Patient education and a collaborative healthcare approach were essential for effective management.

## Discussion

Pseudolesions that are challenging to differentiate from liver malignancies or metastases in imaging techniques often arise from two primary factors. These include temporary extrinsic compression, frequently induced by the diaphragm or ribs, and a distinctive third inflow of blood from nontraditional venous sources. ALGV is the cause of this rare third inflow, with a prevalence of 4% [2].

Third inflow, a crucial concept in hepatic circulation, signifies additional blood supply pathways that supplement the primary arterial and portal venous sources. These supplementary pathways, known as third inflow, play a vital role in maintaining adequate liver perfusion. Particularly in scenarios where portal venous flow is compromised, such as portal vein thrombosis, third inflow becomes highly relevant. It ensures continued blood supply to the liver tissue, contributing significantly to overall hepatic function and viability. Recognizing the significance of third inflow provides a comprehensive understanding of hepatic hemodynamics and its implications, especially for conditions like liver pseudotumors associated with anomalous venous drainage [3].

In our case, the discrimination between pseudolesions attributed to ALGV and authentic liver lesions is accomplished by recognizing the distinct anatomical position and evident visualization of these atypical veins traversing regions characterized by heightened contrast. ALGV is classified into three distinct types. Type 1, functions as a pure accessory portal vein, branching out and flowing through the sinusoids. Type 2 vein exhibits a parenchymatous distribution with anastomosis to the portal vein, while Type 3 vein takes a more cranial course with anastomosis to intrahepatic portal vein branches [4]. In our case, ALGV corresponds to Type 1(Figures 1 and 2).

## Conclusions

This case report highlights the need to recognize and understand third inflow pathways like ALGV for accurate differentiation between pseudolesions and genuine liver lesions. Awareness of these vascular anomalies aids in precise diagnosis and reduces unnecessary invasive procedures, enhancing patient care. Further research is required to better grasp these complex vascular dynamics and their connection to pseudolesions with anomalous venous drainage.
